# A limit on the extent to which increased egg size can compensate for a poor postnatal environment revealed experimentally in the burying beetle, *Nicrophorus vespilloides*

**DOI:** 10.1002/ece3.1876

**Published:** 2015-12-29

**Authors:** Matthew Schrader, Rachel M. Crosby, Aimee R. Hesketh, Benjamin J. M. Jarrett, Rebecca M. Kilner

**Affiliations:** ^1^ Department of ZoologyUniversity of CambridgeDowning StreetCambridgeCB2 3EJU.K; ^2^ Department of BiologyUniversity of the SouthSewaneeTennessee37383

**Keywords:** Burying beetle, egg size, *Nicrophorus vespilloides*, parental care

## Abstract

It is often assumed that there is a positive relationship between egg size and offspring fitness. However, recent studies have suggested that egg size has a greater effect on offspring fitness in low‐quality environments than in high‐quality environments. Such observations suggest that mothers may compensate for poor posthatching environments by increasing egg size. In this paper we test whether there is a limit on the extent to which increased egg size can compensate for the removal of posthatching parental care in the burying beetle, *Nicrophorus vespilloides*. Previous experiments with *N. vespilloides* suggest that an increased egg size can compensate for a relatively poor environment after hatching. Here, we phenotypically engineered female *N. vespilloides* to produce large or small eggs by varying the amount of time they were allowed to feed on the carcass as larvae. We then tested whether differences between these groups in egg size translated into differences in larval performance in a harsh postnatal environment that excluded parental care. We found that females engineered to produce large eggs did not have higher breeding success, and nor did they produce larger larvae than females engineered to produce small eggs. These results suggest that there is a limit on the extent to which increased maternal investment in egg size can compensate for a poor posthatching environment. We discuss the implication of our results for a recent study showing that experimental *N. vespilloides* populations can adapt rapidly to the absence of posthatching parental care.

## Introduction

Parents influence the phenotype and fitness of their offspring through both the genes that they transmit to them (i.e., heredity) and the environments they provide for them (i.e., parental effects). For many years, evolutionary biologists focused primarily on heredity's role in shaping offspring phenotype and the importance of parental effects was marginalized (Wade 1989). This perspective has shifted somewhat and there is now great interest in the ecological and evolutionary consequences of parental effects (Badyaev and Uller 2009).

Perhaps the most common and well‐studied parental effect in animals is egg size. A positive association between egg size and offspring fitness is often assumed to exist (Smith and Fretwell 1974), and there is general support for this assumption in vertebrates and invertebrates, with many studies finding that smaller eggs have lower hatching success and produce lower fitness hatchlings than larger eggs (Roff 1992; Czesak and Fox 2003). In some species, however, the relationship between egg size and offspring fitness depends on the quality of the environment experienced by offspring, with a stronger relationship between egg size and offspring fitness occurring in low‐quality environments than in high‐quality environments (Fox 2000; Czesak and Fox 2003; Bashey 2006). Consistent differences between populations in environmental quality can lead to evolutionary divergence in egg size, with selection favoring larger eggs or newborns in poor quality environments and smaller eggs or newborns in high‐quality environments (Hutchings 1991; Rollinson and Hutchings 2013). When environmental quality varies predictably within a population (either spatially or temporally), selection may favor mothers that plastically modify their investment in eggs in response to the quality of the environment their offspring will develop in (Fox et al. 1997; Koenig et al. 2009). Adaptive divergence in egg size and adaptive plasticity in egg size can both be thought of as means by which mothers can compensate for the negative impact of poor quality environments on offspring fitness.

Although most studies analyzing the compensatory effects of egg size in poor environments have focused on physical or ecological attributes of the environment (Fox 2000; Bashey 2006; Rollinson and Hutchings 2013), social aspects of the environment experienced by offspring may also play a role in determining the relationship between egg size (or size at birth) and offspring fitness. In animals that care for their young after birth or hatching, parents themselves are an important component of the social environment that their offspring will experience (Moore et al. 1997; Wolf and Brodie 1998; Lock et al. 2004) and variation in the quality or quantity of care that parents provide may change the relationship between egg size and offspring fitness. For example, in a facultative cooperative breeder, the superb fairy wren, *Malurus cyaneus*, the effect of egg size on offspring phenotype is contingent on the posthatching social environment that chicks experience. Cooperatively breeding females produce smaller eggs than pair‐breeding females, but the negative effects of a small egg size are compensated by the additional posthatching care provided by helpers. Conversely, pair‐breeding females lay larger eggs, but their offspring gain no advantage because they are tended only by the female and her male, and so receive less provisioning after hatching (Russell et al. 2007, 2008). More recently, Monteith et al. (2012a) have shown that in the burying beetle, *Nicrophorus vespilloides*, the relationship between egg size and larval fitness is similarly sensitive to the social environment experienced by larvae. When *N. vespilloides* parents are allowed to provision their young, there is no relationship between egg size and larval survival or growth rate. However, when parents were prevented from caring for their young, there was a positive relationship between egg size and larval growth rate, which is likely to be correlated with fitness (Monteith et al. 2012a).

Here, we are interested in understanding whether there is a limit on the extent to which an increased egg size can compensate for a poor postnatal environment. The experiment we describe here is part of a wider research program in our laboratory that investigates whether contrasting types of parental care yield contrasting forms of evolutionary change. As part of this work, we have recently shown that laboratory populations of *N. vespilloides* adapt to experimentally altered regimes of posthatching care: populations raised without posthatching care for generation after generation evolved to fare better in this environment than populations where parental care is present after hatching (Schrader et al. 2015a).

However, it is unclear which traits contribute to this adaptation. One possibility is that adaptation to the absence of posthatching care has involved an evolutionary increase in egg size. Indeed, studies such as those described above suggest that the harsh posthatching environments could give rise to selection for increased egg size. It might be thought that previous work by Monteith et al. (2012a) renders another experiment to test this possibility redundant. However, there are key differences between the protocol for removing postnatal care in our long‐term evolution experiments (Schrader et al. 2015a) and that used by Monteith et al. (2012a), which make the environment experienced by larvae immediately after hatching in our experiments far harsher than that experienced by larvae in the work by Monteith et al. (details are given in the Methods below). We were therefore interested to test whether an increase in egg size can still be compensatory even in the very harsh postnatal conditions created in our experimental evolution study, or whether there is a limit on the capacity for compensation in this way.

The logic of our experiment was to test whether an increase in egg size could compensate for a harsh postnatal environment within a single generation of a new population of *N. vespilloides*, as a first step toward pinpointing the adaptations we generated separately in different populations undergoing experimental evolution (Schrader et al. 2015a). The ideal test of this hypothesis would involve directly measuring the relationship between egg size and breeding success under the same “no care” environment used in our previous study (Schrader et al. 2015a). Unfortunately, this is not possible, as measuring individual eggs requires searching through the soil for eggs, removing the eggs, and then measuring them (either weighing them or photographing them to make linear measurements). This process is quite invasive and was not performed under the “no care” conditions in our previous study, so we avoided doing it here. Instead, our approach was to phenotypically engineer females to produce eggs of different sizes (Steiger 2013). We then tested whether the resulting differences in egg size influenced breeding success and larval performance in the absence of posthatching care. One potential drawback of this approach is that manipulating female size may alter variables other than egg size. For example, Steiger (2013) has shown that female body size influences larval mass in *N. vespilloides* and that this effect is likely due to larger females providing better posthatching parental care. However, such size‐related posthatching parental effects are not an important source of phenotypic variation in our study since posthatching care was completely eliminated (see below).

## Methods

### Phenotypic engineering

The beetles used in this experiment were descended from field‐collected beetles trapped in 2014 from two sites in Cambridgeshire, UK. These beetles were interbred with a laboratory population for several generations prior to our experiments. We began by creating females that differed in adult body size by manipulating the amount of time they were allowed to feed on the breeding carcass as larvae (i.e., using a technique developed by Steiger 2013). We bred 40 pairs of beetles (all of which had been reared with full parental care), placing each pair in a box with soil and a thawed mouse carcass (21–25 g). These boxes were then put in a dark cupboard to simulate underground conditions. Five days after pairing, we removed approximately 10 larvae from each family (early larvae). Each larva was placed within a cell (individual cell dimensions: 2 cm × 2 cm × 1.8 cm) in an eclosion box (box dimensions, length × width × depth: 10 cm × 10 cm × 1.8 cm), covered with damp peat, and left to pupate. The remaining larvae (Late larvae) in each box were left to feed with full access to parental care for three more days, after which they were removed and placed in eclosion boxes.

After pupation, we haphazardly collected four newly eclosed females (two early females and two late females) from each family. We then photographed these beetles and placed them individually in boxes (box dimensions, length × width × depth: 12 cm × 8 cm × 2 cm) with a small amount of ground beef and some soil. We measured the size of each female (pronotum width in mm) from the digital photographs. Each female was housed individually and fed ground beef twice per week until they were bred 14 days after eclosion (see below). This protocol resulted in females that differed significantly in body size (mean pronotum width of early females ± 1 SEM = 4.44 ± 0.050 mm, *n* = 53; mean pronotum width of late females ± SEM = 5.25 ± 0.033 mm, *n* = 66; *t* = 13.46, *P *<* *0.0001).

### Experiment 1: The relationship between body size and investment in eggs

We first tested whether the body size variation we had created resulted in differences between the early and late treatments in clutch size and egg mass. To do this, we mated 20 females from each treatment group with an unrelated male from our stock population. These pairs were mated as described above; however, we used smaller carcasses (10–16 g) to be consistent with our previous work (Schrader et al. 2015a,b). Fifty‐three hours after pairing these individuals, we removed the parents and counted the number of eggs that had been laid in the soil (clutch size). We then combined and weighed ten eggs chosen haphazardly from each clutch to estimate mean egg mass. We were not able to weigh ten eggs from six clutches because the eggs ruptured. These clutches were evenly divided between the two treatments and were excluded from all analyses. In addition, a female in the early treatment failed to produce any eggs and was subsequently dropped from the analyses.

We first statistically tested whether female size was correlated with both clutch size and egg mass (pooling the early and late females). Next, we tested whether the early and late females displayed differences in mean clutch size and mean egg mass. Neither clutch size nor mean egg mass varied with the mass of the breeding carcass (linear regression of clutch size on carcass mass: *R* ^2^ *= *−0.017, *P *=* *0.50, *n* = 33; linear regression of mean egg mass on carcass mass: *R* ^2^ = −0.03, *P *=* *0.99, *n* = 33). Some families (10 of 40) were represented by females in both the early and late treatment groups. To account for this, we initially tested whether there were differences between the early and late females in mean clutch size and mean egg mass using separate linear mixed effect models where female family was included as a random effect. Female family was not significant in any model (clutch size, χ^2^ = 0.206, *P *=* *0.65; mean egg mass, χ^2^ = 0, *P *=* *1), and the results of mixed models including female family are the same as simpler nonparametric comparisons that exclude this effect (Mann–Whitney *U*‐tests). For the sake of simplicity, we reported the results of the Mann–Whitney *U*‐tests.

### Experiment 2: Does egg mass influence breeding success in the absence of posthatching parental care?

We next tested whether egg mass influenced breeding success in the absence of posthatching parental care. For this experiment, we used the same “no care” environment used by Schrader et al. (2015a), which is different from the posthatching environment used by Monteith et al. (2012a) in two important ways. First, Schrader et al. (2015a) eliminated posthatching parental care by removing parents 53 h after pairing. This is after the female has completed the clutch but before the eggs have hatched (Boncoraglio and Kilner 2012) and is also before parents create a shallow depression in the carcass for newly hatched larvae to feed from. In contrast, Monteith et al. (2012a) removed parents at larval hatching (around 72 h after pairing). By this time, parents have created a feeding depression for larvae to feed in. Second, Schrader et al. (2015a) did not manipulate the carcass in any way after the parents were removed. In contrast, Monteith et al. (2012a) cut a small hole in the carcass when the parents were removed. As a consequence of these differences, the no care environment used by Schrader et al. (2015a) is likely more challenging for newly hatched larvae than the no care environment used by Monteith et al. (2012a).

We mated females from each treatment group (early and late) with unrelated males from our stock population, using the procedure described above. Fifty‐three hours after pairing these individuals, we removed both parents from the breeding box. Eight days after pairing, we scored breeding success and measured the number and size of larvae in each successful attempt. Breeding success was scored as a binomial response: breeding attempts with at least one dispersing larva were scored as successes and attempts with no dispersing larva were scored as failures. We tested whether breeding success (the proportion of breeding attempts producing at least one dispersing larva) differed between the early and late treatments using a χ^*2*^ test. For successful broods, we also tested whether brood size at dispersal and the average mass of dispersing larvae were affected by the female's treatment. Brood size was not influenced by carcass mass (linear regression of brood size at dispersal on carcass mass, *R* ^2^ = −0.029, *P *=* *0.73, *n* = 31), and nor was it normally distributed, so we used a Mann–Whitney U‐test to compare brood size between the two treatments. We performed an additional analysis of brood size combining failed and successful breeding attempts. For this, we assigned failed breeding attempts a brood size of 0 and use a GLM with a Poisson error term corrected for overdispersion to compare brood size between the two maternal treatment groups. Mean larval mass was not influenced by carcass mass (linear regression of mean larval mass on carcass mass, *R* ^2^ = −0.03, *P *=* *0.75, *n* = 31), so we did not include carcass mass as a covariate in our analysis of mean larval mass. Mean larval mass varied with brood size but in a nonlinear manner (see below). To account for this, we initially compared larval mass between the two treatments using a linear mixed effect model including treatment as a factor, brood size and brood size^2^ as covariates (see Schrader et al. 2015b for similar analyses), and female family as a random effect. Preliminary analyses found no effect of female family on mean larval mass (χ^2^ = 2.56, *P *=* *0.11). Furthermore, there were no differences between the early and late treatments in the shape of the relationship between brood size and mean larval mass, as indicated by nonsignificant interactions between treatment and brood size (*P *=* *0.93) and treatment and brood size^2^ (*P = *0.95). Below we report the results of the analysis excluding female family and these interaction terms.

## Results

### Experiment 1: The relationship between body size and investment in eggs

**Figure 1:**
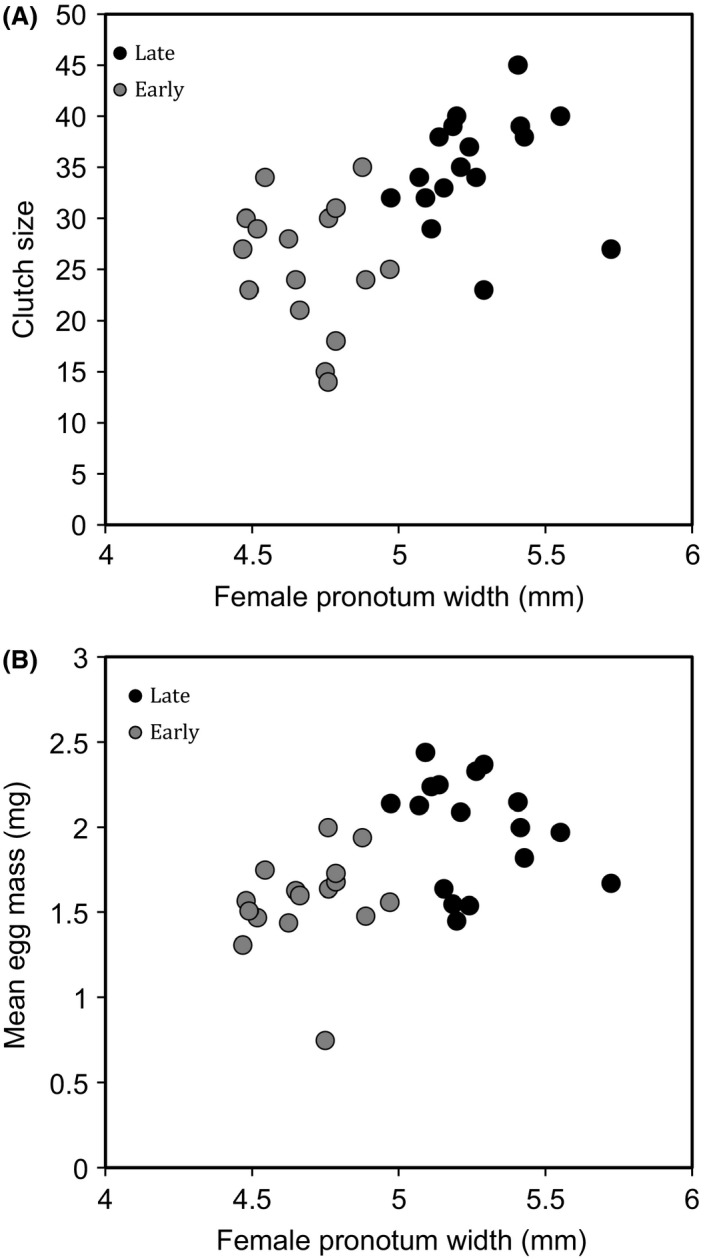
The relationships between female size (pronotum width in mm) and clutch size (A) and female size and mean egg mass (in mg) (B). As larvae, late females (black circles) were allowed to feed on the carcass for longer than early females (gray circles).

The subset of females that were used to examine the relationship between body size and clutch size differed in body size, with early females being significantly smaller than late females (mean pronotum width ± 1 SEM: early = 4.69 ± 0.039 mm, late = 5.26 ± 0.046 mm, *t* = 9.42, *P *<* *0.0001, *n* = 33). Pooling early and late females, there were significant positive correlations between female size and clutch size (*r *=* *0.54, *P *=* *0.0011, *n* = 33, Fig. 1A), and female size and mean egg mass (*r *=* *0.47, *P *=* *0.0056, *n* = 33, Fig. 1B). On average, early females had significantly smaller clutches and lighter eggs than late females (mean clutch size ± 1 SEM: early = 25.5 ± 1.56, late = 35 ± 1.32, W = 238, *P *=* *0.00025; mean egg mass ± 1 SEM: early = 1.566 ± 0.070 mg, late = 1.99 ± 0.077 mg, *t* = 4.04, *P *<* *0.0001). Given that female size affected both clutch size and egg mass, it is not surprising that clutch size and mean egg mass were positively correlated with one another (pooling early and late females, *r *=* *0.363, *P *=* *0.038*, n* = 33).

### Experiment 2: Does egg mass influence breeding success in the absence of posthatching parental care?

Experiment 1 confirmed that early and late females differ in the number of eggs that they produce and the average size of those eggs. We next asked whether these differences influenced maternal breeding success. In this experiment, early females were again significantly smaller than late females (mean pronotum width of early females = 4.33, *n* = 30, mean pronotum width of late females = 5.32 mm, *n* = 30; *t* = 12.58, *P *<* *0.0001). However, we found no evidence that female treatment affected maternal breeding success: 47% of the breeding attempts involving an early female were successful, and 60% of the breeding attempts involving a late female were successful (χ^2^ = 0.603, *P *=* *0.44). Among the females that bred successfully, we also found no evidence that female treatment affected the number of larvae produced (mean early = 11.36, *n* = 14; mean late 11.33, *n* = 18; W = 113.5, *P *=* *0.65). Combining failed and successful broods into a single analysis yielded similar results to the separate analyses described above: There was no difference between the early and late treatments in the number of larvae produced (*t *=* *−0.597, *P *=* *0.55, dispersion parameter = 15.55).

**Figure 2:**
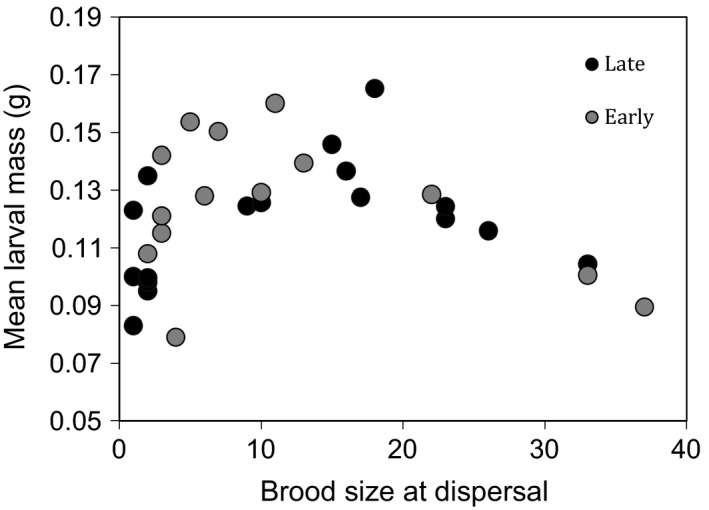
The relationship between brood size at dispersal and mean larval mass (g). Black and gray circles indicate broods with late and early mothers, respectively. All broods were sired by fathers from a stock population.

As in a previous study (Schrader et al. 2015a), mean larval mass varied with brood size in a nonlinear manner and was best described by a quadratic regression (Table 1, Fig. 2). However, there was no significant difference between the early and late treatments in mean larval mass (effect of treatment, *P *=* *0.17; Table 1, Fig. 1).

**Table 1:** Results of a general linear model examining the effects of treatment (early vs. late), brood size, and brood size^2^ on average larval mass. Preliminary analyses found no significant interactions between brood size and treatment or brood size^2^ and treatment, and these interactions were dropped from the final model

Factor	*F*_1, 28_	*P*
Treatment	1.95	0.17
Brood size	23.57	<0.0001
Brood size^2^	25.87	<0.0001

## Discussion

The benefits of producing large eggs often vary with the environmental conditions that young experience after hatching. In benign environments, there is often no relationship between egg size and offspring fitness, whereas in harsh environments, offspring fitness increases with egg size. In this experiment, we used phenotypic engineering to create female *N. vespilloides* that produced small or large eggs and then tested whether the egg size differences we created influenced offspring survival and growth in a harsh environment lacking posthatching parental care. We found that females engineered to produce large eggs did not have significantly higher breeding success or produce larger offspring in the absence of care than females engineered to produce smaller eggs. These results suggest that there is a limit on the extent to which increased egg size can compensate for the removal of posthatching care in *N. vespilloides*.

Our experiment is similar to a recent study by Monteith et al. (2012a) who measured the impact of egg size on larval survival and mass at dispersal in two social environments (with care and without care). Although both studies found no relationship between egg size and breeding success in the absence of posthatching care, our study had a lower breeding success rate than Monteith et al.'s (see Table 2). Our results also differ from Monteith et al.'s with respect to the effect of egg size on larval mass at dispersal. While Monteith et al. (2012a) found a positive relationship between egg size and larval mass at dispersal in the absence of care, we found no relationship between these two variables.

**Table 2:** Measures of larval performance in the absence of parental care in this study and a similar study conducted by Monteith et al. (2012a). Breeding success is the proportion of breeding attempts that produced at least one dispersing larva. Mean brood size is the average number of dispersing larvae in successful broods. Mean larval mass is the average mass in grams of larvae from the successful broods. Data from this study are pooled across the early and late female treatments. Data from Monteith et al. are from the absence of parental care treatment and were obtained from the Dryad Digital Repository (Monteith et al. 2012b). All measurers of larval performance are lower in this study than in Monteith et al. (2012a, 2012b)

	This study	Monteith et al. (2012a)
Breeding success (%)	53 (*n* = 60)	62.5 (*n* = 40)
Mean brood size	11.34 (*n* = 32)	20.12 (*n* = 25)
Mean larval mass (g)	0.121 (*n* = 32)	0.134 (*n* = 25)

We recognize three potential explanations for the differences between these two studies. First, they may be due to differences in the way that egg size was measured. We weighed a sample of eggs from each clutch to determine the relationship between maternal size and mean egg mass, whereas Monteith et al. (2012a) measured the length and width of a sample of eggs from each clutch and estimated average egg volume based on these measurements. Unfortunately, we do not have estimates of both egg volume and egg mass for the females in our study, so we cannot test whether larger eggs are indeed heavier. However, egg mass and egg volume are both positively correlated with female size in *N. vespilloides* (this study and others), suggesting that the late females in this study likely produced eggs with a larger volume than the early females.

A second possible explanation for the differences between these two studies is the experimental or statistical approach used to look for an association between egg size and larval performance (breeding success and mass). To test whether egg size influences larval survival and mass, we compared breeding success and larval mass between groups engineered to produce large or small eggs. In contrast, Monteith et al. (2012a) tested whether naturally occurring, continuous variation in average egg volume was correlated with breeding success and larval mass. We do not believe that differences between the way data were analyzed in the two studies explains the difference in results as there is no evidence in our study that continuous variation in female size (which is strongly correlated with egg size) influences either breeding success (logistic regression of breeding success on female pronotum width: *P *=* *0.452) or average larval mass (linear regression of mean larval mass on female pronotum width: *P *=* *0.139). Furthermore, our phenotypically engineered females fell within the range of sizes seen in natural populations of *N. vespilloides*, and the experimental populations that were the subject of our previous work (Schrader et al. 2015a).

The third, and in our minds the most likely explanation, is that major differences between the two studies in the duration of prehatching care and the treatment of the carcass after parental removal account for the difference in results. We removed parents 53 h after pairing, which is after the clutch is complete but before the eggs hatch, and before parents create a depression on the surface of the carcass that newly hatched larvae feed from. We did not manipulate carcasses after parents were removed. In contrast, Monteith et al. (2012a) removed parents 72 h after pairing, which is around the time eggs hatch and parents have typically made a feeding depression by this time. In addition, Monteith et al. (2012a) made an incision in the carcass when parents were removed to allow larvae to access the carcass. As a result of these differences, the no care environment in our study is probably much harsher for newly hatched larvae than Monteith et al.'s (2012a). This is supported by the higher rate of total brood loss, smaller average brood sizes, and lower average larval mass at dispersal in our study compared to Monteith et al.'s (2012a, 2012b) (see Table 2).

Our study is relatively unusual in reporting a limit on the capacity for egg size to compensate for harsh postnatal conditions, so it is worth considering why we found this result. One possibility is that the harsh conditions after hatching that we created experimentally would never be experienced by larvae in nature. Although *N. vespilloides* larvae can survive with no posthatching care, perhaps they are never left alone prior to incisions being made in the carcass. Consequently, perhaps there has been no selection on egg size to compensate for such a harsh posthatching environment, and our experimental manipulation was never going to be capable of phenotypically engineering an egg capable of such a feat. Perhaps the conditions simulated by the Monteith et al. (2012a) study more closely simulate natural conditions than those created by our experiment and this is why they were able to detect a compensatory effect. Unfortunately, too little is known about the natural history of *N. vespilloides* to determine whether this possibility is plausible.

Alternatively, perhaps the primary function of egg size in *N. vespilloides* is not to compensate for a poor postnatal nutritional environment, although it may serve this function incidentally under some conditions. By analogy, egg size laid by shiny cowbirds *Molothrus bonariensis* does not function primarily to compensate for the variable posthatching environment that arises by virtue of this generalist brood parasite exploiting diverse host species. Instead, egg size is selected by hosts that reject any odd‐sized eggs that are laid in their nests (Tuero et al. 2012) and its primary function is to enable this brood parasite to evade this line of host defense. In *N. vespilloides*, egg size might function primarily to confer resistance to desiccation (Jacobs et al. 2013). Casual observation suggests that humidity levels in our laboratory breeding boxes are generally high, whether or not parents provide postnatal care. As our manipulation did not create conditions under which eggs might desiccate, this could explain why we were unable to find a corresponding benefit associated with larger eggs. Further work is needed to investigate this possibility.

In a previous study, Schrader et al. (2015a) found that *N. vespilloides* populations can rapidly adapt to the experimental removal of posthatching care and suggested two mechanisms that might confer this adaptation. First, populations may adapt to a change in posthatching parental care by shifting investment from the posthatching period to the prehatching period. Second, there may have been a change in larval morphology or behavior that enhances larval survival in the absence of care. Our results demonstrate that a simple change in one component of prehatching care (egg size) cannot compensate for the removal of posthatching parental care. Thus, the adaptation observed by Schrader et al. (2015a) is probably not simply due to a change in maternal investment in egg size, unless egg size rapidly increased beyond the upper limits observed in natural populations. Alternative possibilities are that adaptation to the no care regime has involved a change in some other component of prehatching parental care (e.g. carcass preparation, antimicrobial exudate activity), a change in the nutritional content of eggs (independent of size), or a change in larval behavior or morphology. We are currently examining these possibilities.

## Conflict of Interest

None declared.

## References

[ece31876-bib-0001] Badyaev, A. V. , and T. Uller . 2009 Parental effects in ecology and evolution: mechanisms, processes and implications. Philos. Trans. R. Soc. Lond. B Biol. Sci. 364:1169–1177. 19324619 10.1098/rstb.2008.0302 PMC2666689

[ece31876-bib-0002] Bashey, F. 2006 Cross‐generational environmental effects and the evolution of offspring size in the Trinidadian guppy *Poecilia reticulata* . Evolution 60:348–361. 16610325

[ece31876-bib-0003] Boncoraglio, G. , and R. M. Kilner . 2012 Female burying beetles benefit from male desertion: sexual conflict and counter‐adaptation over parental investment. PLoS ONE 7:e31713. 22355390 10.1371/journal.pone.0031713 PMC3280230

[ece31876-bib-0004] Czesak, M. , and C. Fox . 2003 Evolutionary ecology of egg size and number in a seed beetle: genetic trade‐off differs between environments. Evolution 57:1121–1132. 12836828 10.1111/j.0014-3820.2003.tb00321.x

[ece31876-bib-0005] Fox, C. W. 2000 Natural selection on seed‐beetle egg size in nature and the laboratory: variation among environments. Ecology 81:3029–3035.

[ece31876-bib-0006] Fox, C. W. , M. S. Thakar , and T. A. Mousseau . 1997 Egg size plasticity in a seed beetle: an adaptive maternal effect. Am. Nat. 149:149. 10.1111/j.1558-5646.1999.tb03790.x 28565419

[ece31876-bib-0007] Hutchings, J. A. 1991 Fitness consequences of variation in egg size and food abundance in brook trout *Salvelinus fontinalis* . Evolution 45:1162–1168. 10.1111/j.1558-5646.1991.tb04382.x 28564166

[ece31876-bib-0008] Jacobs, C. G. C. , G. L. Rezende , G. E. M. Lamers , and M. van der Zee . 2013 The extraembryonic serosa protects the insect egg against desiccation. Proc. Biol. Sci. 280:20131082. 23782888 10.1098/rspb.2013.1082 PMC3712428

[ece31876-bib-0009] Koenig, W. D. , E. L. Walters , and J. Haydock . 2009 Helpers and egg investment in the cooperatively breeding acorn woodpecker: testing the concealed helper effects hypothesis. Behav. Ecol. Sociobiol. 63:1659–1665. 19701482 10.1007/s00265-009-0773-y PMC2728902

[ece31876-bib-0010] Lock, J. E. , P. T. Smiseth , and A. J. Moore . 2004 Selection, inheritance, and the evolution of parent‐offspring interactions. Am. Nat. 164:13–24. 15266367 10.1086/421444

[ece31876-bib-0011] Monteith, K. M. , C. Andrews , and P. T. Smiseth . 2012a Post‐hatching parental care masks the effects of egg size on offspring fitness: a removal experiment on burying beetles. J. Evol. Biol. 25:1815–1822. 22775779 10.1111/j.1420-9101.2012.02567.x

[ece31876-bib-0012] Monteith, K. M. , C. Andrews , and P. T. Smiseth . 2012b Data from: post‐hatching parental care masks the effects of egg size on offspring fitness: a removal experiment on burying beetles. Dryad Digital Repository. doi:10.5061/dryad.fj472. 10.1111/j.1420-9101.2012.02567.x 22775779

[ece31876-bib-0013] Moore, A. , E. III Brodie , and J. Wolf . 1997 Interacting phenotypes and the evolutionary process: I. Direct and indirect genetic effects of social interactions. Evolution 51:1352–1362. 10.1111/j.1558-5646.1997.tb01458.x 28568644

[ece31876-bib-0014] Roff, D. A. 1992 The evolution of life histories theory and analysis. Chapman and Hall, New York, NY.

[ece31876-bib-0015] Rollinson, N. , and J. A. Hutchings . 2013 Environmental quality predicts optimal egg size in the wild. Am. Nat. 182:76–90. 23778228 10.1086/670648

[ece31876-bib-0016] Russell, A. , N. Langmore , A. Cockburn , L. Astheimer , and R. Kilner . 2007 Reduced egg investment can conceal helper effects in cooperatively breeding birds. Science 317:941–944. 17702942 10.1126/science.1146037

[ece31876-bib-0017] Russell, A. F. , N. E. Langmore , J. L. Gardner , and R. M. Kilner . 2008 Maternal investment tactics in superb fairy‐wrens. Proc. Biol. Sci. 275:29–36. 17956851 10.1098/rspb.2007.0821 PMC2562397

[ece31876-bib-0018] Schrader, M. , B. J. M. Jarrett , and R. M. Kilner . 2015a Using experimental evolution to study adaptations for life within the family. Am. Nat. 185:610–619. 25905504 10.1086/680500 PMC4497813

[ece31876-bib-0019] Schrader, M. , B. J. M. Jarrett , and R. M. Kilner . 2015b Parental care masks a density‐dependent shift from cooperation to competition among burying beetle larvae. Evolution 69:1077–1084. 25648525 10.1111/evo.12615 PMC4476075

[ece31876-bib-0020] Smith, C. , and S. Fretwell . 1974 The optimal balance between size and number of offspring. Am. Nat. 108:499–506.

[ece31876-bib-0021] Steiger, S. 2013 Bigger mothers are better mothers: disentangling size‐related prenatal and postnatal maternal effects. Proc. R. Soc. B Biol. Sci. 280:20131225. 10.1098/rspb.2013.1225 PMC3730594 23843390

[ece31876-bib-0022] Tuero, D. T. , V. D. Fiorini , B. Mahler , and J. C. Reboreda . 2012 Shiny cowbird *Molothrus bonariensis* egg size and chick growth vary between two hosts that differ markedly in body size. J. Avian Biol. 43:227–233.

[ece31876-bib-0023] Wade, M. J. 1989 The evolutionary genetics of maternal effects Pp. 5–21 *in* Mousseau T. A. and Fox C. W., eds. Maternal effects as adpataitons. Oxford Univ. Press, New York, NY.

[ece31876-bib-0024] Wolf, J. , and E. III Brodie . 1998 The coadaptation of parental and offspring characters. Evolution 52:299–308. 10.1111/j.1558-5646.1998.tb01632.x 28568322

